# Unravelling the dynamics of genotype and environment interactions on chilli (*Capsicum annuum* L.) yield-related attributes in soilless planting systems

**DOI:** 10.1038/s41598-023-50381-0

**Published:** 2024-01-19

**Authors:** Siti Sahmsiah Sahmat, Mohd Yusop Rafii, Yusuff Oladosu, Mashitah Jusoh, Mansor Hakiman, Hasmah Mohidin

**Affiliations:** 1 Institute of Tropical Agriculture and Food Security, Universiti Putra Malaysia, 43400 Serdang, Malaysia; 2 Faculty of Plantation and Agrotechnology, Universiti Teknologi MARA, Cawangan Sarawak, 94300 Kota Samarahan, Malaysia; 3 Faculty of Plantation and Agrotechnology, Universiti Teknologi MARA Jasin, 77300 Merlimau, Malaysia; 4 Department of Crop Science, Faculty of Agriculture, Universiti Putra Malaysia, 43400 Serdang, Malaysia; 5 Bioresource Management (BIOREM), Institute of Tropical Forestry and Forest Products, (INTROP), Universiti Putra Malaysia, 43400 Serdang, Malaysia

**Keywords:** Plant breeding, Plant genetics

## Abstract

Evaluation of genotypes to identify high-yielding and stable varieties is crucial for chilli production sustainability and food security. These analyses are essential, particularly when the breeding program aims to select lines with great adaptability and stability. Thirty chilli genotypes were evaluated for yield stability under four soilless planting systems viz; fertigation, HydroStock (commercial hydrogel), BioHydrogel (biodegradable hydrogel), and hydroponic to study the influence of genotype by environment interaction. The research used a split-plot randomized complete block design (RCBD) with two cropping cycles and five replications. The GGE biplot analysis was employed to assess the mean versus stability perspective in explaining the variation in genotypic and genotype-by-environment effects on the yield-related attributes for yield per plant, fruit number, fruit length, and width. Stability analysis denoted genotypes G26 and G30 as the most stable for yield per plant, while G16, G22, and G30 were stable for the number of fruits per plant. Among the four planting systems evaluated, HydroStock and BioHydrogel outperformed the others in yield per plant, demonstrating the highest level of informativeness or discrimination. These findings offer critical insights for future crop breeding programs and the optimization of agricultural practices.

## Introduction

Chilli (*Capsicum annuum* L.) holds immense value among various crops due to its wide consumption, versatile usage, and crucial role in culinary traditions worldwide. However, enhancing the yield remains challenging as conventional approaches to analyzing the genotype performance often overlook the significant effects and interactions that profoundly influence crop yield and selection^1^. The productivity and quality of chili pepper are influenced by agronomic factors, which are subjected to the impacts of cultivar selection and environmental variability across diverse environments. The genotype × environment interaction (G × E), a phenomenon where the response of a plant variety varies depending on several factors such as soil type, temperature, irrigation management, photoperiod, and pathogenic disease^2–4^ is also known as genotype-by-environment interaction (GEI). This GEI involves variations in genetic makeup that affect traits or gene expression levels in different environmental conditions^5^ or specialized for specific planting conditions^6^, posing challenges for plant breeders in introducing new cultivars and recommending superior genotypes. Concurrently, stability analysis emerges as an equally vital tool, assessing the adaptability of genotypes across various environments and contributing to a more comprehensive understanding of crop performance^7–9^ emerges as an equally vital tool, assessing the adaptability of genotypes across various environments and contributing to a more comprehensive understanding of crop performance^5,10^. The screening and selection of cultivars by plant breeders frequently involve the utilization of yield performance and phenotypic expression. Therefore, the primary focus of crop technology is to mitigate risk, promote yield stability, reduce costs, and increase profitability^7,11^.

Incorporating soilless planting systems into this framework is increasingly important. Soilless cultivation offers controlled environments that can mitigate some of the variability and unpredictability associated with traditional soil-based agriculture^12^. These systems allow for a more precise study of G × E interactions by minimizing external environmental variables, leading to a clearer understanding of genetic influences on yield and quality. Furthermore, soilless systems can be key in developing and selecting cultivars that are not only high-yielding but also resilient to a range of environmental conditions addressing issues of food security and sustainable agricultural practices^13^.

The analysis of variance (ANOVA) is a widely used statistical technique to analyze multi-environmental yield trials, aiming to detect genotype-by-environment interaction by assessing the variation among random and fixed factors such as replication, genotype, site, season, and year. However, it is important to note that ANOVA has certain limitations when detecting genotypic differences in non-additive expression, commonly referred to as G × E interaction^14^. Numerous statistical approaches have been developed to assess genotype stability, capturing different aspects of genotype-by-environment interaction. These methods can help identify genotypes that exhibit consistent performance across different environments which includes the stability analysis techniques deviation from regression (S^2^_d_), regression slope (b_i_), Wricke ecovalance (*Wi*^*2*^), and Shukla stability variance (*σi*^*2*^). Stability under a broad range of environmental conditions is observed when the genotypes’ regression coefficient (b_i_) approach unity in conjunction with a high trait mean.

Over the last three decades, several statistical techniques have been devised to evaluate the consistency of cultivars across different testing locations. Among the most crucial analytical techniques, biplot analysis provides a comprehensive graphical tool for visualizing and assessing G × E interactions in plant breeding and agricultural research to help researchers identify genotypes with stable performance across diverse environments and select superior cultivars. Biplot analysis has proven valuable in various crops, including cereals, legumes, vegetables, and fruits, enabling breeders to make informed decisions in cultivar development and selection^15–17^. Incorporating G × E data into biplots, researchers gain a deeper understanding of the complex interplay between genotypes and environments, leading to improved breeding strategies, interest trait improvement, and the development of resilient cultivars. AMMI (Additive Main Effects and Multiplicative Interaction) biplots and GGE (Genotype plus Genotype by Environment) biplots are the two types of biplot analysis frequently utilized^18–20^. The AMMI biplot visually represents the relationships between genotypes, environments, and the G × E interaction patterns. In an AMMI biplot, genotypes are represented as vectors, similar to other biplots. The vectors’ length and direction indicate the genotypes’ performance and direction. The GGE model characterizes the Genotype by Environment (G × E) interaction as a composite of the genotype’s main effect and the interaction between the genotype and environment. The GGE biplot identifies mega-environments, groups of similar environments where genotypes perform similarly. This help determines the ideal genotypes for specific mega-environments and assesses the adaptability and stability of genotypes across these mega-environments^21^. Therefore, this study aims to determine the stability analysis of yield and yield attributes in different soilless planting systems of chilli.

Conversely, genotypes displaying low mean performance for a trait signify inadequate adaptation to all environments. Furthermore, genotypes exhibiting a b_i_ value exceeding unity demonstrate a heightened sensitivity to environmental fluctuations, indicative of below-average stability, while showcasing a more pronounced adaptability to conditions favoring high yields. The assessment of genotype stability involves examining the statistical significance of deviation from regression (S^2^_d_). Genotypes characterized by low Wricke ecovalance (*Wi*^*2*^) and Shukla stability variance (*σi*^*2*^) are considered stable, indicating their reliability in various conditions.

## Materials and methods

### Planting materials

The seeds from Chilli Bangi 3 (B3) and Chilli Bangi 5 (B5) were initially acquired from the Seeds Company, Bangi, Selangor, Malaysia. These seeds were then subjected to gamma irradiation in the greenhouse facility at the Malaysian Nuclear Agency Bangi, 43000 Kajang, Selangor, Malaysia, utilizing Caesium-137 (^137^Cs) as a source of chronic gamma irradiation^22^ to obtain a M_1_ parent. The commercial varieties (Co), on the other hand, were obtained from local agricultural supplier companies in Seri Kembangan, Selangor as detailed in Table 1. For this study, a total of 19 mutant lines (M_6_ generation) and 11 commercial genotypes were selected to undergo trials in four soilless planting systems: fertigation, HydroStock (commercial hydrogel), BioHydrogel (biodegradable hydrogel), and a hydroponic in two planting cycles (2020–2022). The irrigation system was installed in the rain shelter following MARDI’s System Manual (first edition). The plants were manually supplied daily with a copper standard formulation fertilizer containing (mg L^−1^) Mo 0.2, N 200, P 60, Ca 170, K 300, Mg 50, Mn 2, Fe 12, B 1.5, Zn 0.1, and Cu 0.1, with electron conductivity (EC) readings ranging from 0.6 to 2.5 according to the growth stage.

**Table 1:** List of chilli genotypes used in the study.

Commercial	Mutant	
G8-CO-V828	G1-C-B3-C0	G13-C-B3R14
G19-CO- Local	G2-C-B3-100Gy	G14-C-B5C0
G20-CO-V826	G3-C-B3-200Gy	G15-A-B5100Gy
G21-CO-Local	G4-C-B3-300Gy	G16-A-B5200Gy
G22-CO-V104	G5-C-B3R2	G17-A-B5300Gy
G23-CO-Local 116	G6-C-B3R3	G18-A-B5400Gy
G26-CO-V108	G7-C-B3R4	G24-C-B5R7
G27-CO-Local 461	G9-C-B3R7	G25-C-B5R8
G28-CO-Local	G10C-B3R8	
G29- CO-VE015	G11-C-B3R9	
G30- CO-VE017	G12-C-B3R11	

### Field experiments

The research was organized using a split-plot Randomized Complete Block Design (RCBD), with each planting system replicated five times across two cropping cycles. The experiment was conducted under an experimental glasshouse located at Field 15, Universiti Putra Malaysia Serdang, with an average temperature ranging from 25 to 33 °C, average humidity of 75%.

#### Preparation of soilless planting systems

For the fertigation system, the 16″ × 16″ polybag was filled with cocopeat, ensuring adequate space at the top for watering and further planting procedures. Seedlings were then planted in the cocopeat within the polybag by making a small hole in its centre, placing the seedling inside, and gently covering it with the medium. Following planting, the polybag was positioned in an area that provided the plants with the requisite sunlight.

The process for preparing the commercial hydrogel known as HydroStock was adhered to as per the supplier's instructions. An amount of 20 g of the dry hydrogel was combined with 1.5 L of water. After a swelling period of 30 min, the hydrogel was ready to be incorporated with cocopeat.

The solidified BioHydrogel was soaked in distilled water with initial pH of 12–13 and electrical conductivity over 20.00 mScm^−1^. It was kept at room temperature and the water was changed daily for 6–10 days until the pH reached a neutral 7.0–7.5 and conductivity fell to 0.2–0.5 mScm^-1^. The time needed for neutralization varied based on the hydrogel's size and water volume. Post-neutralization, the hydrogel was crushed into smaller fragments, each 2–5 mm in diameter, to maximize surface area, preparing it for use as an amendment in cocopeat for field deployment.

The Nutrient Film Technique hydroponic system, established in a complex glasshouse, was organized using a randomized complete block design. Seedlings, aged 21 days, were transplanted into the system, ensuring that all roots were submerged in the nutrient solution. Throughout the growth period, the Electrolytic Conductivity (EC) of the solution was carefully maintained between 0.5 and 2.0 Ms cm^-1^. Oxygenation was provided by pumps attached to the nutrient tank, enhancing aeration. To ensure optimal nutrient availability, the nutrient stock in the tank was refreshed every time an adjustment in EC levels was made.

### Stability analysis

The G × E interaction was examined utilizing the R-studio software^23–25^. The output from the G × E analysis contained univariate stability results, including the Linn and Binn (Pi), regression coefficient (b_i_), deviation from regression (S_d_^2^), Shukla stability variance (σ^i^_2_), Wricke’s ecovalance (W_i_^2^), adjustments in trait means (M) and least significant differences (L.S.D.) across the planting cycles and systems for the genotype. A GGE biplot and AMMI were employed for multivariate stability to interpret the G × E interaction visually. This approach incorporates two main components, the GGE (Genotype and Genotype by Environment) and the biplot^26,27^, and was analyzed using R-studio software packages of ggplot2 and Agricolae^23,25^.

### Complies with international, national and/or institutional guidelines

Experimental research and field studies on plants (either cultivated or wild), comply with relevant institutional, national, and international guidelines and legislation.

## Results

### “Which won where” polygon view of GGE biplot

**Figure 1:**
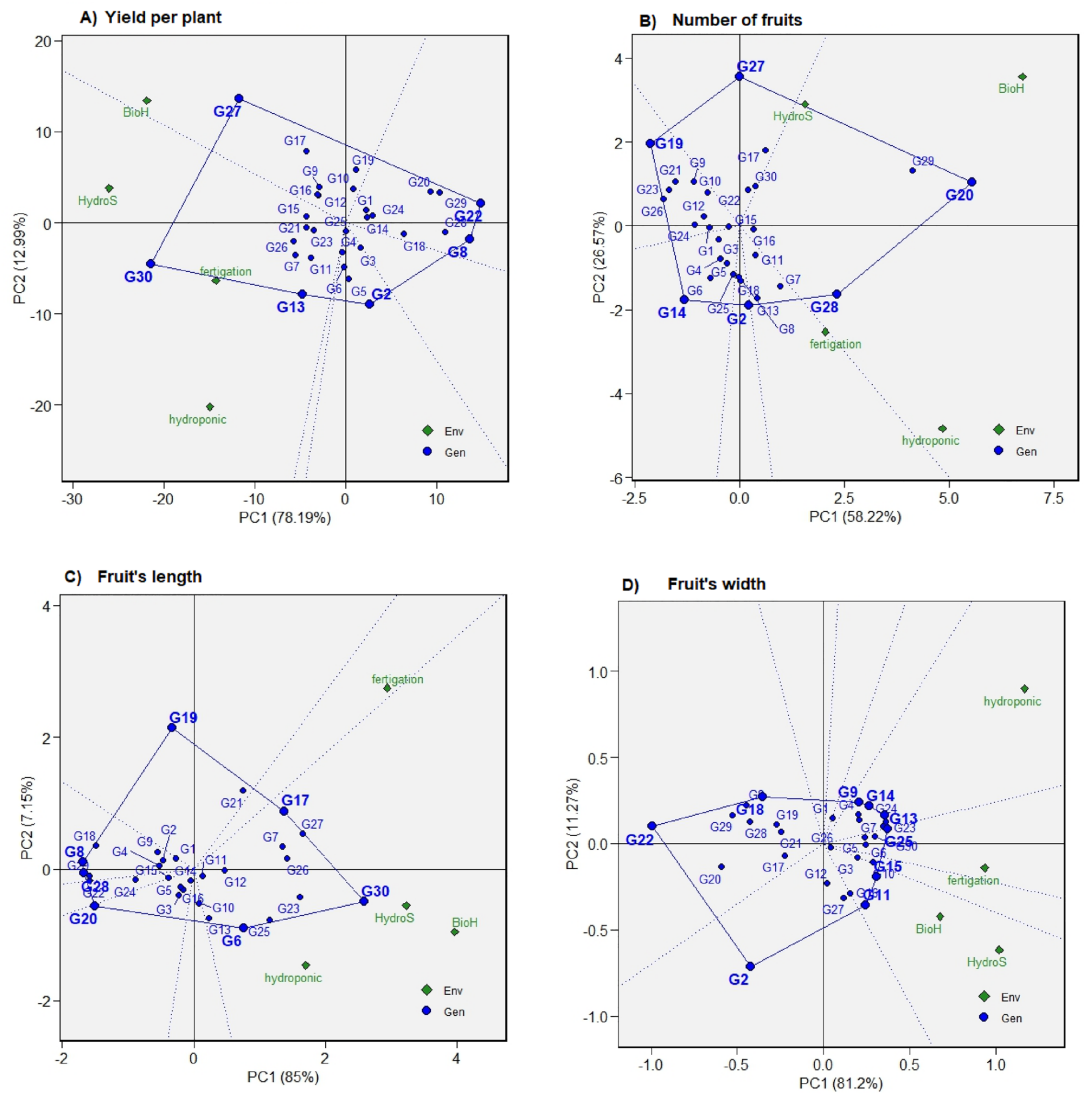
Polygon view of GGE biplot (which–won–where) showing the (G + G × E) interaction effect of 30 chilli genotypes within four planting systems for (**A**) yield per plant, (**B**) the number of fruits, (**C**) Fruit’s length, (**D**) Fruit’s width. The biplot was based on Scaling = 0, Centering = 2, S.V.P. = 3. Bio = BioHydrogel, Hydros = HydroStock.

The polygon view of the GGE biplot (Fig. 1) demonstrates significant variation in the genotype and genotype-by-environment interaction, accounting for 91%, 85%, 92%, and 93% of the variation in the yield per plant (g), number of fruits, fruit length, and fruit width respectively. The environmental markers are segmented into single, two, two, and three sectors for these traits, with unique genotypes taking the lead in each section. This pattern affirms the presence of gene-environment interactions across all the traits studied. Genotypes located at the vertex of sectors containing environmental markers indicate superior performance in yield and adaptability in those specific environments. However, the genotypes at the vertex, where no environment falls within the sector, demonstrated consistently inferior performance across all environments. Conversely, the genotypes within the polygon exhibited less responsiveness to the environment than the corner genotypes, indicating that the corner genotypes possess a superior adaptive capability across diverse environments compared to those within the polygon.

### Polygon view of AMMI2 biplot

**Figure 2:**
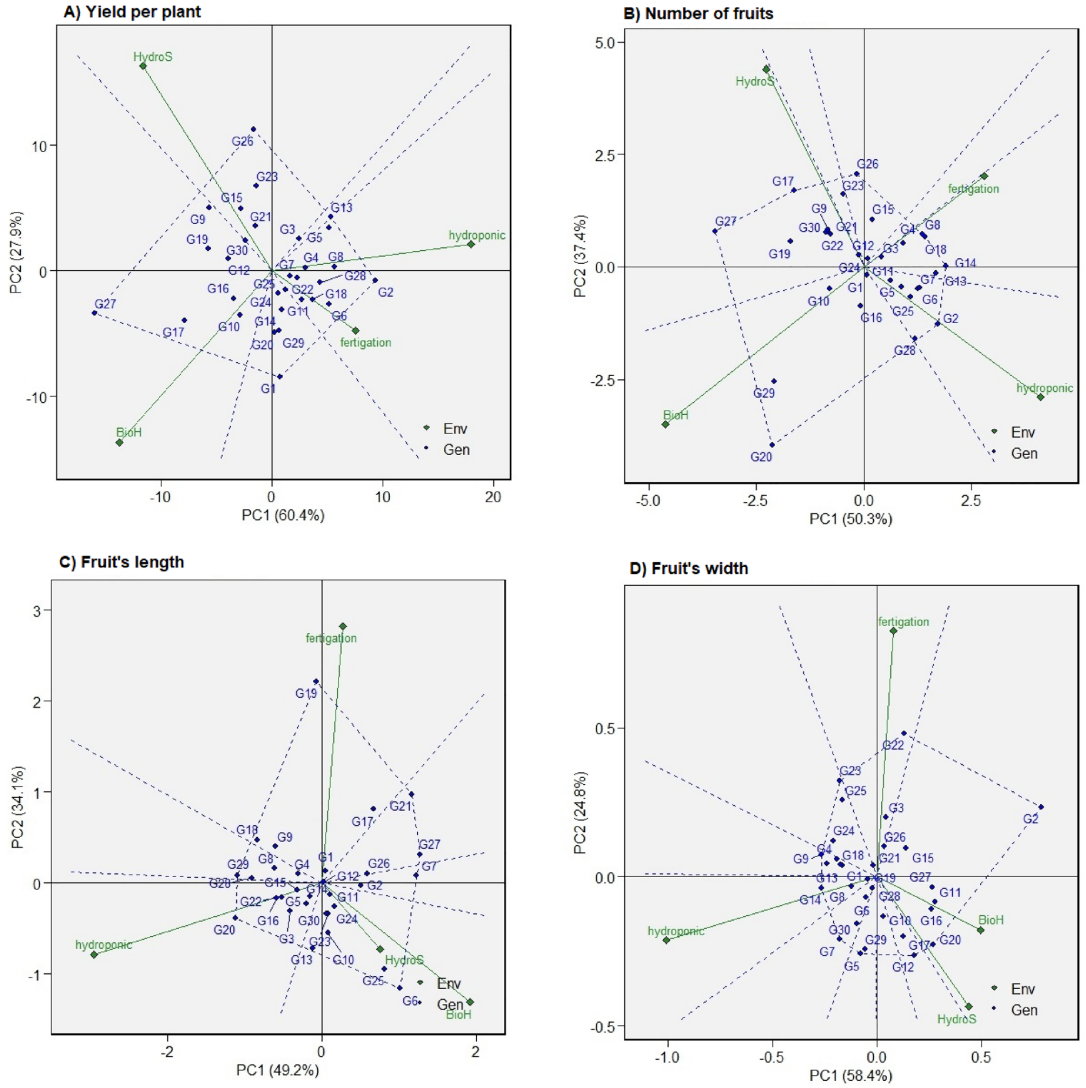
Polygon view of Additive main effects and multiplicative interaction 2 (AMMI2) biplot of the first two principal components (PC1 and PC2) showing the (G + G × E) interaction effect of 30 chilli genotypes in two planting cycles and four planting systems for (**A**) Yield per plant, (**B**) Number of fruits, (**C**) Fruit’s length, (**D**) Fruit’s width. The biplots were based on Scaling = 0, Centering = 0, and SVP = 2.

The AMMI2 analysis is a valuable graphical instrument that utilizes the information in the first two principal components (PC1 and PC2) to unravel the gene-environment interactions (GEIs), including characterizing the major environments and pinpointing genotypes that exhibit broad or specific adaptability. The distribution of the first two principal component interactions in this model account for 88.3%, 87.7%, 83.3%, and 83.2% of the variation in yield per plant, number of fruits, fruit length, and width, respectively, further substantiating the occurrence of GEI (Fig. 2A–D). These figures suggest that the primary two PCs effectively predicted the interactions among the 30 chilli genotype trials conducted across four different environmental systems, thus, providing a comprehensive understanding of how genotypes perform in varying environments. These suggested that the first two PCs of genotypes predicted the interaction of the 30 chilli genotypes trials in four environment systems.

In Fig. 2, the biplot can be divided into four distinct sectors by vertical and horizontal lines that pass through the center (0, 0). Similar to the GGE biplot approach, the genotypes positioned at the polygon’s vertices are identified as the winning cultivars within the specific sectors associated with the respective environments. The spatial arrangement of the environment and genotype vectors, originating from the central point of the biplot diagram, provides a visual representation of the dynamic interaction between the environment and genotypes. The further these vectors extend from the origin, the more significant the magnitude of this interaction is likely to be. This study revealed that the sectors corresponding to yield per plant, number of fruits, fruit length, and width consist of five, seven, six, and nine distinct segments, respectively, with different genotypes winning each sector.

### Genotype evaluation

The significant G × E interaction in this current study justified our evaluation of chilli genotypes for yield-attribute stability across the planting systems (Table 2). The result of the mean comparison presented in Table 3 showed that genotypes G30, G27, and G7 had the highest yield per plant, while genotypes G22 and G8 had the lowest yield per plant. While for the number of fruits, genotypes G20 and G29 recorded the highest number of fruits and contrary to G19 and G21, which revealed the lowest number of fruits.

**Table 2:** Analysis of variance (mean square) for yield attributes on 30 *Capsicum annuum* L. genotypes.

Source	DF	Yield per plant (g)	Weight per fruit (g)	Fruit length (cm)	Fruit width (mm)
Cycle (C)	1	375,087.92***	8.58*	5.77	0.38***
Rep (Cycle)	8	47,414.68***	1.01	7.60***	0.03
System (S)	3	922,391.68***	265.01***	824.50***	6.41***
Cycle*System	3	302,294.76***	16.65***	14.92***	0.46***
System*Rep (Cycle)	24	46,334.28***	2.23^ns^	2.70^ns^	0.09
Genotype (G)	29	813,243.37***	657.76***	453.72***	4.63***
C × G	29	20,300.93***	2.62	5.52***	0.08
S × G	87	95,108.31***	21.70***	39.47***	0.42***
C × S × G	87	13,533.14*	2.17 ns	6.16***	0.08***

*SOV* source of variation, *DF* degrees of freedom.

***Significance at the 0.001 level.

**Significance at the 0.01 level.

*Significance at the 0.05 level.

**Table 3:** Mean comparison of yield attributes on 30 *Capsicum annuum* L. genotypes within different soilless systems. The means followed by similar letters within the column for each parameter were not significantly different at P ≤ 0.05 based on the LSD multiple mean comparisons.

Genotype	Yield per plant (g)	Weight per fruit (g)	Fruit length (cm)	Fruit width (mm)
G1	412.03^kl^	11.83^h–j^	9.91^j–l^	1.84^fg^
G2	419.9^jkl^	10.79^k^	9.04^m^	1.47^ij^
G3	417.43^jkl^	11.33^jk^	10.25^i–k^	2.01^c–e^
G4	465.63^g–j^	12.37^gh^	9.26^lm^	2^c–e^
G5	450.28^h–k^	12.08^hi^	10.21^i–k^	2.04^b–e^
G6	471.51^f–i^	13.44^ef^	12.63^f^	2.04^b–e^
G7	564.41^c^	13.06^f^	14.49^de^	2d^e^
G8	185.53^o^	4.51^n^	5.73^p^	1.37^jk^
G9	484.43^e–h^	14.1^d^	9.35^lm^	2d^e^
G10	425.18^i–l^	12.36^gh^	10.94^gh^	2.08^a–d^
G11	534.63^cde^	13.31^f^	11.17^gh^	2.06^a–d^
G12	496.14^e–h^	14.1^d^	12.27^f^	1.82^g^
G13	553.88^cd^	13.32^f^	11.53^g^	2.13^ab^
G14	409.98^kl^	12.99^f^	10.7^hi^	2.05^b–d^
G15	523.83^cde^	13.18^f^	9.71^k–m^	2.13^ab^
G16	503.94^d–g^	12.87^fg^	10.54^h–j^	1.99^de^
G17	520.76^c–f^	11.76^h–j^	14.89^cd^	1.61^h^
G18	333.81^m^	8.25^l^	6.51^o^	1.46^ij^
G19	404.15^kl^	13.99^de^	9.86^kl^	1.55^hi^
G20	262.93^n^	4.6^mn^	6.57^o^	1.26^l^
G21	526.06^c–e^	16.42^b^	12.61^f^	1.57^h^
G22	150.85^o^	3.71^o^	6.04^op^	0.86^m^
G23	508.71^d–g^	15.4^c^	15.83^b^	2.12^ab^
G24	392.77^l^	11.53^ij^	7.86^n^	2.14^ab^
G25	451.83^h–k^	12.32^gh^	14.03^e^	2.16^a^
G26	547.66^cd^	16.35^b^	15.00^cd^	1.87^fg^
G27	640.79^b^	16.55^b^	15.43^bc^	1.94^ef^
G28	239.13^n^	5.13^m^	5.95^op^	1.4^j^
G29	244.44^n^	4.66^mn^	6.31^op^	1.29^kl^
G30	874.93^a^	21.3^a^	18.95^a^	2.1^a–c^
LSD	45.11	0.60	0.60	0.09

#### Genotype stability and interaction of chilli genotype for yield and yield attributes (Univariate stability methods)

For the stability analysis and the G × E interaction, the chilli genotypes are evaluated based on the yield per plant, number of fruits, fruit length, and width, which showed a positive relationship with the yield per plant. The genotypes with the lowest *P*_*i*_ value are considered the most superior. Hence the genotype with low *P*_*i*_ and high mean yield is considered stable and can be ranked from G30 > G27 > G7 > G13 > G26 > G21 > G11 > G15 > G17 > G16. Genotypes with large *P*_*i*_ values are considered unstable, as recorded by G22, G8, G28, G29, and G20. Genotypes with small W_i_ values are less affected by the environmental or planting system. The genotypes with small values were ranked as follows: G22 > G8 > G29 > G28 > G20 > G18 > G19 > G2 > G1 > G14. The most unstable genotype for yield per plant was G30, G7, G21, G13, and G11 (Table 4). For the number of fruits, genotypes with low *P*_*i*_ and high mean for the number of fruits are ranked from G20 > G29 > G28 > G7 > G17 > G30 > G22 > G16 > G13 > G8. Genotypes with large *P*_*i*_ stability values are considered unstable, as recorded by G19, G26, G14, G23, and G21. The genotypes with small W_i_ values for the number of fruits were ranked as G19 > G26 > G27 > G14 > G21 > G23 > G9 > G10 > G1. The most unstable genotype for the number of fruits were G20, G29, G28, G7, and G30 (Table 5). The genotype with low *P*_*i*_ and high mean for fruit’s length is ranked as G30 > G23 > G26 > G27 > G25 > G7 > G6 > G12 > G21 > G13. While genotypes with large *P*_*i*_ stability values are considered unstable, as recorded by G8, G28, G22, G29, and G18. The genotypes with small W_i_ values for the fruit’s length were ranked as follows: G28 > G29 > G20 > G8 > G22 > G18 > G24 > G19 > G2. The most unstable genotype for the fruit’s length was G30, G23, G26, G27, and G17 (Table 6). The stable genotype with low *P*_*i*_ and high mean for fruit’s width is ranked from G25 > G24 > G13 > G15 > G23 > G30 > G10 > G6 > G14 > G3. While genotypes with large *P*_*i*_ stability values are considered unstable, as recorded by G22, G20, G29, G2, and G8. The genotypes with small W_i_ values for the fruit’s width were ranked as follows: G22 > G2 > G20 > G29 > G8 > G28 > G18 > G17 > G19 > G21. The most unstable genotype for the fruit’s width were G15, G25, G30, G10, and G24 (Table 7).

**Table 4:** Means (corrected by least squares) (M), Linn and Bin (Pi), Regression coefficient (b_i_), Shukla’s stability variance (σ^2^_i_), Wricke’s ecovalence (W_i_^2^), deviation from regression (S^2^_d_), and Annicchiarico (W_i_) yield per plant planted in 4 different planting systems.

GEN	Mean	*P*_*i*_	W_i_^2^	σ^2^_i_	W_i_	b_i_	S^2^_d_
G1	412.03	118,995.20	186,046.60	12,949.37	76.90	0.94	15,957*
G2	419.90	122,512.90	337,764.60	23,786.37	73.41	− 1.15	9907
G3	417.43	112,340.00	48,192.10	3102.62	84.96	0.59	1413
G4	465.63	92,647.95	39,918.23	2511.63	97.03	0.08	− 2562
G5	450.28	101,189.00	161,556.20	11,200.05	85.29	0.04	9317
G6	471.51	93,680.71	116,914.80	8011.39	93.34	− 0.48	− 1052
G7	564.41	53,745.85	37,893.66	2367.02	117.87	0.86	1076
G8	185.53	252,763.60	118,337.90	8113.04	33.93	− 0.57	− 2175
G9	484.43	81,939.85	176,206.90	12,246.54	90.88	2.58	3531
G10	425.18	107,253.80	71,628.41	4776.64	83.82	1.90	783
G11	534.63	65,516.40	85,053.24	5735.56	107.82	0.65	5312
G12	496.14	76,671.79	60,775.77	4001.45	100.89	1.91	− 392
G13	553.88	62,029.29	150,062.00	10,379.04	109.12	− 0.23	5380
G14	409.98	119,427.10	96,599.99	6560.33	79.51	0.28	4626
G15	523.83	67,398.87	94,899.49	6438.86	105.75	1.59	5245
G16	503.94	73,955.99	54,615.66	3561.45	103.63	1.85	− 469
G17	520.76	73,118.23	306,683.9	21,566.32	93.16	2.59	16,358*
G18	333.81	160,263.30	116,051.1	7949.69	63.76	− 0.38	208
G19	404.15	116,380.40	133,449.70	9192.45	73.07	2.66	− 2006
G20	262.93	197,172.10	60,615.78	3990.03	49.20	0.98	3430
G21	526.06	65,197.80	42,734.11	2712.76	110.03	1.53	351
G22	150.85	273,165.20	20,083.30	1094.85	32.52	0.36	− 2533
G23	508.71	74,249.88	125,120.50	8597.50	101.10	1.25	9596
G24	392.77	124,795.60	26,873.02	1579.83	81.68	0.60	− 662
G25	451.83	96,711.96	11,520.79	483.24	97.24	0.63	− 2113
G26	547.66	64,628.14	327,164.40	23,029.21	102.14	1.34	29,549*
G27	640.79	45,758.14	954,496.10	67,838.62	104.22	5.34	6142
G28	239.13	215,011.20	73,436.14	4905.77	47.54	− 0.24	− 2400
G29	244.44	209,604.10	59,117.03	3882.97	45.91	0.71	2900
G30	874.93	216.84	43,400.20	2760.34	191.17	1.80	− 1208

**Table 5:** Means (corrected by least squares) (M), Linn and Bin (Pi), Regression coefficient (b_i_), Shukla’s stability variance (σ^2^_i_), Wricke’s ecovalence (W_i_^2^), deviation from regression (S^2^_d_), and Annicchiarico (W_i_) the number of fruits planted in 4 different planting systems.

GEN	Mean	*P*_*i*_	W_i_^2^	σ^2^_i_	W_i_	b_i_	S^2^_d_
G1	35	460.61	514.7	34.63	80.46	− 0.98	− 3.16
G2	39	389.28	1171.75	81.56	85.45	− 1.87	28.3
G3	37	419.34	70.77	2.92	91.59	0.19	− 21.7
G4	38	419.2	283.32	18.1	90.64	0.27	0.53
G5	37	419.39	371.51	24.4	86.8	0.90	13.5
G6	35	477.28	465.1	31.09	81.69	− 0.2	11.60
G7	44	281.37	513.47	34.54	102.32	− 0.02	19.50
G8	41	375.82	670.28	45.74	93.53	− 1.33	0.49
G9	34	506.62	842.24	58.02	74.09	3.99	− 10.1
G10	34	464.41	231.6	14.41	79.68	1.61	− 3.35
G11	41	327.92	201.55	12.26	97.97	1.02	− 3.42
G12	35	459.41	70.48	2.90	86.57	0.57	− 18.00
G13	42	352.04	937.48	64.83	93.87	0.20	65.20
G14	34	571.16	950.45	65.75	73.19	− 0.8	45.70
G15	40	380.77	269.02	17.08	94.6	1.18	3.09
G16	39	331.13	166.59	9.76	94.45	0.99	− 6.91
G17	44	286.84	1299.59	90.69	96.98	3.14	70.30
G18	41	382.15	1258.42	87.75	89.68	− 2.75	− 9.11
G19	28	666.94	820.00	56.44	58.56	3.27	17.7
G20	58	27.12	4659.96	330.72	118.76	3.58	390***
G21	32	555.40	350.24	22.88	73.44	2.65	− 10.10
G22	41	320.37	317.48	20.54	96.69	1.45	6.61
G23	33	565.22	655.5	44.69	73.53	2.13	31.8
G24	34	497.08	219.21	13.52	81.06	− 0.3	− 15.1
G25	38	402.88	441.71	29.41	88.02	− 0.93	− 8.86
G26	33	586.38	996.83	69.07	71.8	1.99	68.4
G27	38	392.48	3203.44	226.68	72	5.07	166*
G28	48	183.88	1120.85	77.92	109.34	0.58	87.10
G29	54	46.84	2614.68	184.63	115.28	2.44	221**
G30	42	302.87	326.07	21.15	98.7	1.99	1.35

**Table 6:** Means (corrected by least squares) (M), Linn and Bin (P_i_), Regression coefficient (b_i_), Shukla’s stability variance (σ^2^_i_), Wricke’s ecovalence (W_i_^2^), deviation from regression (S^2^_d_), and Annicchiarico (W_i_) for fruit’s length planted in 4 different planting system.

GEN	Mean	*P*_*i*_	W_i_^2^	σ^2^_i_	W_i_	b_i_	S^2^_d_
G1	9.91	42.34	3.45	0.11	88.53	0.95	− 0.14
G2	9.04	50.18	19.24	1.23	73.23	1.68	− 0.46
G3	10.25	38.74	19.59	1.26	87.84	0.66	1.02
G4	9.26	48.15	7.22	0.37	82.54	0.68	− 0.18
G5	10.21	38.79	32.52	2.18	85.31	1.08	2.75
G6	12.63	23.22	141.31	9.95	97.44	2.29	6.83**
G7	14.49	15.59	149.57	10.54	112.54	2.04	10***
G8	5.73	89.58	25.45	1.68	49.56	0.25	− 0.25
G9	9.35	48.30	32.50	2.18	79.51	0.27	0.57
G10	10.94	32.54	36.84	2.49	91.88	1.41	2.53
G11	11.17	31.75	8.32	0.45	98.78	1.00	0.36
G12	12.27	23.23	0.09	− 0.13	112.65	1.01	− 0.47
G13	11.53	28.7	30.1	2.01	98.07	0.90	2.49
G14	10.70	34.83	2.82	0.06	96.50	0.86	− 0.28
G15	9.71	43.52	12.46	0.75	84.52	0.76	0.54
G16	10.54	36.74	23.97	1.57	90.05	0.38	0.32
G17	14.89	12.4	81.42	5.68	123.06	1.46	6.78**
G18	6.51	81.85	75.69	5.27	52.43	− 0.26	0.52
G19	9.86	50.77	269.23	19.09	65.13	0.74	26.2***
G20	6.57	80.00	89.35	6.24	49.21	− 0.30	1.50
G21	12.61	24.81	170.54	12.04	93.15	2.53	6.89**
G22	6.04	86.14	35.12	2.37	50.64	0.20	0.39
G23	15.83	5.19	8.42	0.46	141.70	1.21	0.19
G24	7.86	62.04	27.28	1.81	62.48	1.47	1.33
G25	14.03	14.56	95.39	6.67	114.91	1.97	5.21
G26	15.00	8.78	28.86	1.92	133.14	1.80	− 0.25
G27	15.43	10.52	119.92	8.42	124.31	2.27	4.83
G28	5.95	88.14	64.17	4.44	48.11	− 0.24	− 0.42
G29	6.31	83.84	82.57	5.76	49.12	− 0.40	− 0.32
G30	18.95	0.01	23.45	1.53	167.03	1.33	1.42

**Table 7:** Means (corrected by least squares) (M), Linn and Bin (Pi), Regression coefficient (b_i_), Shukla’s stability variance (σ^2^_i_), Wricke’s ecovalence (W_i_^2^), deviation from regression (S^2^_d_), and Annicchiarico (W_i_) for fruit’s width planted in 4 different planting systems.

GEN	Mean	*P*_*i*_	W_i_^2^	σ^2^_i_	W_i_	b_i_	S^2^_d_
G1	1.84	0.13	0.3	0.02	96.53	0.19	− 0.001
G2	1.47	0.51	4.77	0.34	55.17	4.61	0.05
G3	2.01	0.06	0.2	0.01	106.71	1.44	0.004
G4	2.00	0.07	0.35	0.02	104.41	0.42	0.01
G5	2.04	0.06	0.4	0.03	106.45	0.4	0.02
G6	2.04	0.05	0.1	0.01	109.23	0.55	− 0.01
G7	2.00	0.07	0.44	0.03	103.52	− 0.05	− 0.002
G8	1.37	0.48	0.17	0.01	72.06	0.57	0.00
G9	200	0.07	0.56	0.04	102.65	0.06	0.02
G10	2.08	0.04	0.19	0.01	110.77	0.77	0.01
G11	2.06	0.06	0.79	0.05	104.87	1.79	0.05
G12	1.82	0.17	1.24	0.09	89.59	0.91	0.11
G13	2.13	0.03	0.47	0.03	110.6	− 0.05	0.001
G14	2.05	0.05	0.53	0.04	105.59	− 0.18	− 0.002
G15	2.13	0.03	0.22	0.01	113.78	1.82	− 0.01
G16	1.99	0.08	0.55	0.04	102.31	1.99	0.014
G17	1.61	0.29	0.41	0.03	82.26	1.40	0.025
G18	1.46	0.40	0.41	0.03	74.77	0.56	0.024
G19	1.55	0.32	0.09	0.00	82.69	0.96	− 0.002
G20	1.26	0.62	1.02	0.07	58.21	2.07	0.054
G21	1.57	0.3	0.01	0.00	85.55	0.99	− 0.01
G22	0.86	1.12	1.39	0.1	33.06	2.45	0.061
G23	2.12	0.04	0.91	0.06	108.48	0.48	0.07
G24	2.14	0.03	0.56	0.04	110.7	0.07	0.02
G25	2.16	0.02	0.63	0.04	111.62	0.49	0.04
G26	1.87	0.12	0.16	0.01	99.15	1.46	− 0.001
G27	1.94	0.1	0.67	0.05	98.43	1.85	0.03
G28	1.40	0.45	0.17	0.01	73.03	1.07	0.01
G29	1.29	0.57	0.32	0.02	66.65	0.48	0.013
G30	2.10	0.04	0.20	0.01	111.03	0.44	0.00

#### Genotype ranking

**Figure 3:**
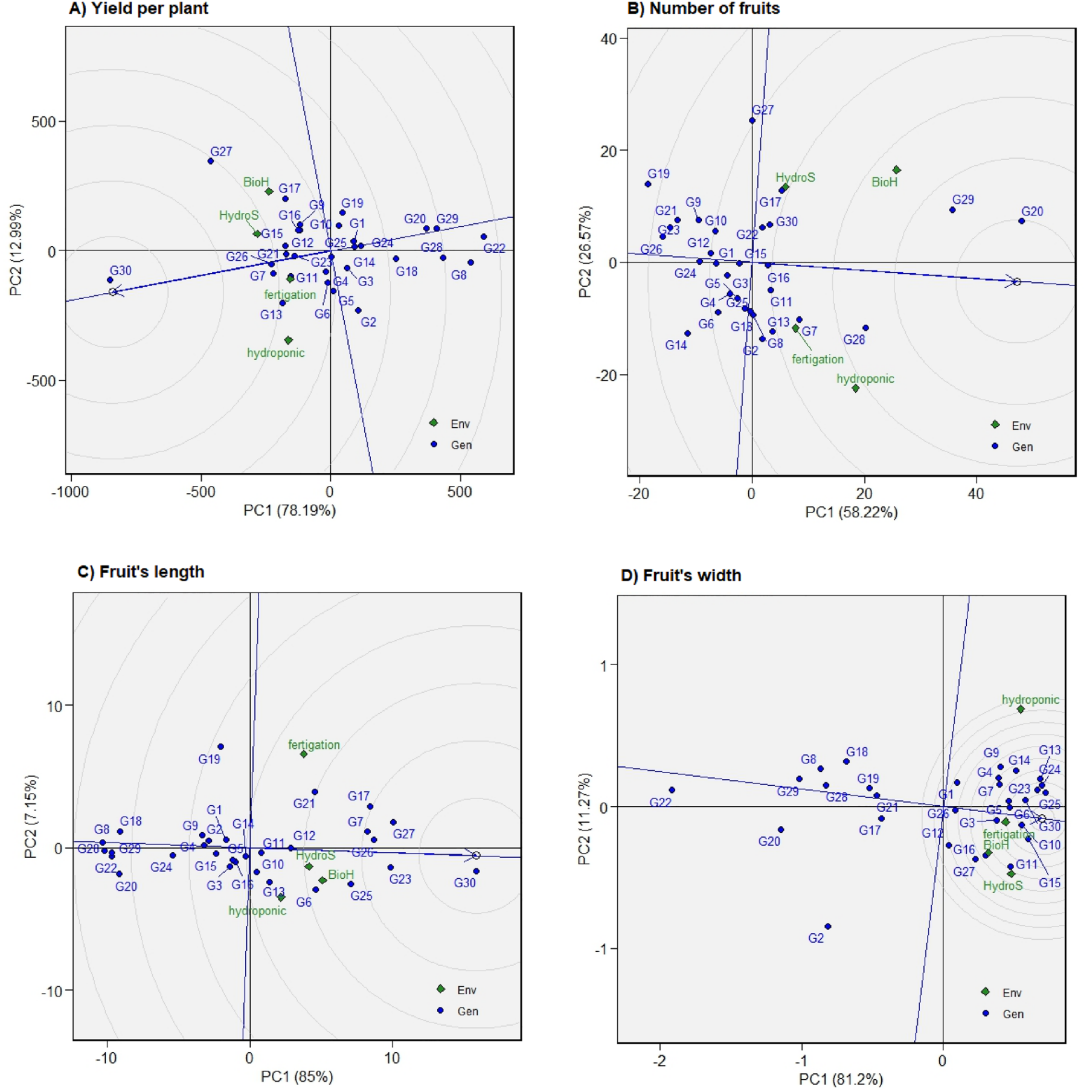
The genotypes comparison with the ideal genotype view showing the (G + G × E) interaction effect of 30 chilli genotypes in two planting cycles and four planting systems (**A**) Yield per plant, (**B**) Number of fruits, (**C**) Fruit’s length, (**D**) Fruit’s width. An ideal genotype is represented by a circle within the innermost concentric circles on average environment coordinate (AEC) –abscissa, which passes through biplot origin.

A biplot with concentric circles presents the genotype ranking or “comparison with ideal genotype”. The ideal genotype always occupies the central position, with the arrowhead in concentric circles (Fig. 3A–D). Those genotypes closer to the center are more desirable than those outside the circle. In the current study, G30 falls within the inner circle, making this genotype suitable for all planting systems environments regarding yield per plant. The G20 is selected for the number of fruits, G30 for the fruit’s length and width. A genotype closer to the ideal genotype is more favorable. Thus, based on proximity to the ideal, the genotype hierarchy for yield per plant is G30 > G26 > G7 > G13 > G27.

#### Mean vs. stability

A mean vs. stability biplot is one way to visualize G × E interactions, often used in plant breeding and genetics. The biplot’s horizontal axis (x-axis) typically represents the genotypes’ mean performance. Genotypes to the right on this axis have higher mean performance across all environments. The vertical axis (y-axis) usually represents stability. Genotypes further from the horizontal axis (either above or below) are less stable, meaning their performance varies more across different environments.

**Figure 4:**
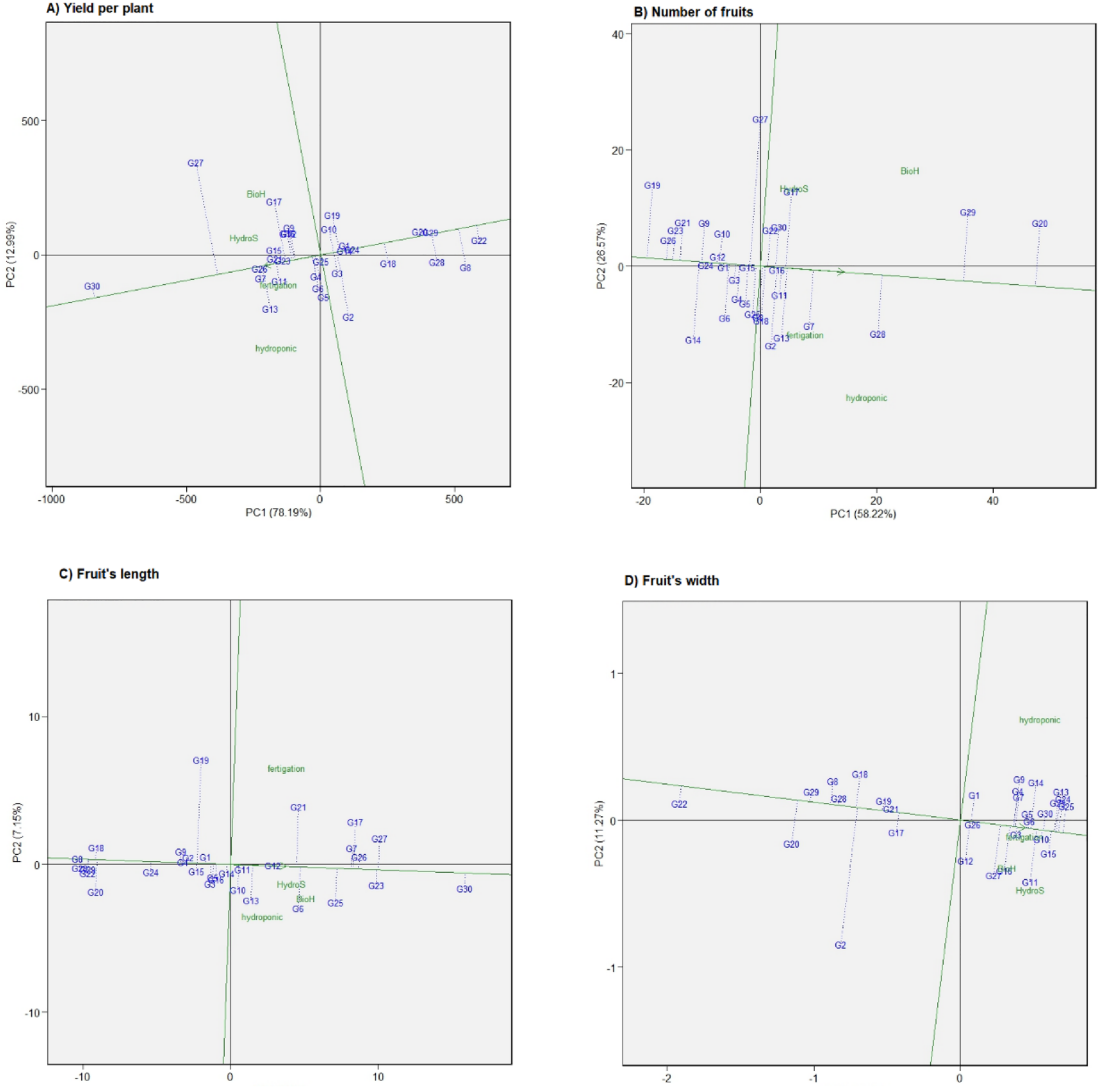
The mean vs. stability view showing the (G + G × E) interaction effect of 30 chilli genotypes in two planting cycles and four planting systems for (**A**) Yield per plant, (**B**) Number of fruits, (**C**) Fruit’s length, and (**D**) Fruit’s width.

Conversely, genotypes close to the horizontal axis are more stable because their performance is consistent across different environments. In this current study, the GGE biplot analysis was employed to assess the mean versus stability perspective in explaining the variation in genotypic and genotype by environment effects for yield-related traits, namely yield per plant, number of fruits, fruit length, and width. The results, illustrated in Fig. 4, indicated that the GGE biplot analysis accounted for substantial proportions of the total variation in these traits, specifically 88%, 84%, 92%, and 94% for yield per plant, number of fruits, fruit length, and width, respectively.

The directional arrows in the biplot provided valuable insights into the ranking of genotypes based on their performance in relation to higher trait values, as indicated on the A.E.C. abscissa. Among the genotypes assessed, G30 exhibited the highest yield per plant, followed by G27, while G8 and G22 demonstrated the lowest yield per plant. Regarding the number of fruits, G20 and G29 displayed the highest fruit yield, whereas G19 exhibited the lowest fruit count. G30 and G27 showed the highest mean values for fruit length, while G13 and G23 exhibited the widest fruit girth among the genotypes. Genotype vectors projected from the AEC vertical axis offer insights into stability, with the most stable genotypes often positioned on the AEC abscissa (horizontal axis) or having the shortest projection from the AEC vertical axis.

Consequently, G26 and G30 emerged as the most stable genotypes for yield per plant, as evidenced by their shortest genotype vectors from the AEC vertical axis. For the number of fruits per plant, G16, G22, and G30 were considered stable genotypes. Similarly, G30, G23, and G26 exhibited stability in terms of fruit length. Finally, G10, G6, and G30 were identified as the most stable fruit-width genotypes. The GGE biplot analysis provided comprehensive insights into the mean versus stability perspective for evaluating genotypic and genotype by environment effects on yield-related traits. The results identified specific genotypes that excelled in trait performance and stability, thereby contributing to the understanding and improving crop breeding programs.

### Additive main effects and multiplicative interaction 1 (AMMI1)

**Figure 5:**
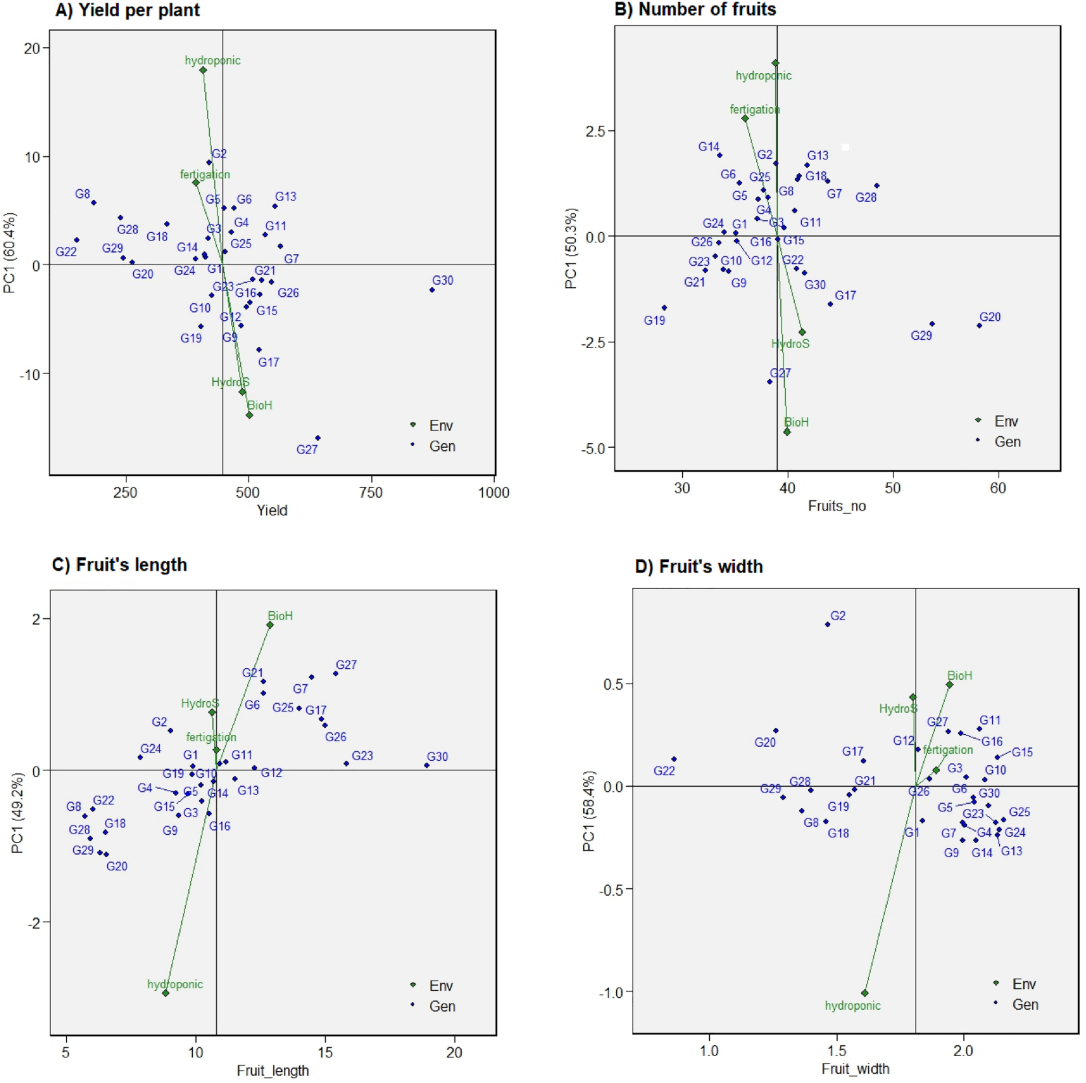
The Additive Main Effects and Multiplicative Interaction 1 (AMMI1) biplot displays the principal components’ impact (PC1) on 30 chilli genotypes across two planting cycles and four systems, showcasing variations in yield per plant (**A**), number of fruits (**B**), fruit length (**C**), and fruit width (**D**).

In the context of AMMI1 analysis, the first principal component (PC1) and the main trait effect are represented by the horizontal (abscissa) and vertical (ordinate) axes of the biplot, respectively. The AMMI model decomposes the effects of genotypes and environments into two primary components: additive effects and multiplicative effects. Figure 5 illustrates that all genotypes exhibited differences not only in their means but also in their interactions. For each yield-related attribute, none of the planting system scores were near zero, indicating the presence of significant interaction between the genotypes and the planting system. These suggest that genotypes are more specific to certain environments or planting systems rather than showing consistent performance across different conditions. However, noteworthy is that genotypes such as G20 and G24 (for yield per plant), G15 and G16 (for the number of fruits), G12, G23, and G30 (for fruit length), and G28 and G21 (for fruit width) displayed nearly zero scores on the first principal component (PCA1), which suggests environmental conditions relatively less influences these genotypes.

Despite their reduced sensitivity to environmental effects, these genotypes exhibited mean values below average for yield per plant. Since plant breeders prioritize genotypes with high yield and relative stability, alternative genotypes must be considered. In this regard, for yield per plant, fruit length, and fruit width, genotypes G26 and G30 were recommended as relatively stable genotypes with nearly general adaptation to various environments Fig. 5.

### Discriminative and representative ability of locations

**Figure 6:**
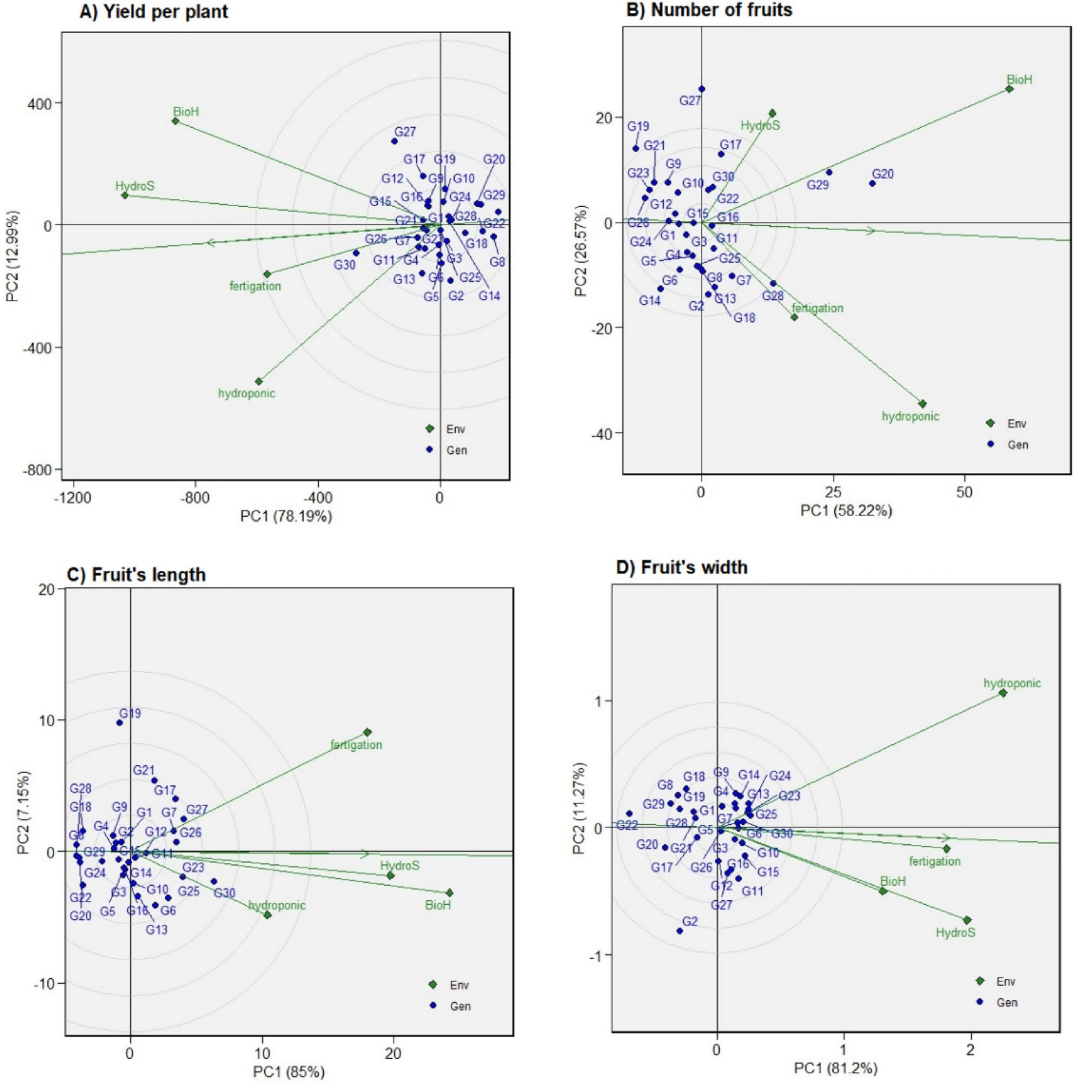
The ‘discriminating power vs. representativeness’ view of the GGE biplot of 30 chilli genotypes planted in four soilless planting systems in two planting cycles of (**A**) Yield per plant, (**B**) Number of fruits, (**C**) Fruit’s length, and (**D**) Fruit’s width.

Figure 6 presents the GGE biplot vector view to determine the discriminative ability of the tested locations. The length of the location vector in the biplot indicates the degree of discriminative ability each location possesses. In the current study, HydroStock and BioHydrogel demonstrated the highest level of informativeness or discrimination among the four planting systems evaluated for yield per plant. In contrast, fertigation and hydroponic systems exhibited lower discriminative abilities (Fig. 6). The representativeness of individual locations was assessed based on the proximate angle between the location vector and the AEC (average environment coordinate). A smaller angle indicates a higher level of representativeness for the tested location. The planting systems amended with hydrogel, specifically BioHydrogel for the number of fruits and HydroStock for the fruit’s length, were identified as the most representative locations within each mega-environment.

## Discussion

This study is the first to document the contribution of genotype and environment to the morpho-physiological traits and yield and yield-related attributes of chilli genotypes across different soilless planting systems. Generally, the yield-related are conducted in different locations to determine the performance of the ideal genotypes in the specific location. The yield from the multi-soilless planting system trial enables quantification of the planting systems and the genotype by environment interaction influences. However, the evaluation of the genotypes and the planting systems or environments cannot be described by analysis of variances solely but require full exploration of G × E that can be achieved through univariate and multivariate analysis^8^. This study offers critical insights into the genotype and genotype-by-environment interaction dynamics of thirty chilli genotypes cultivated across four soilless systems. The observed significant variations in the yield per plant, number of fruits, fruit length, and fruit width, as illustrated by the GGE biplot (Fig. 1), underline the complex interplay between genotypes and their environments^16^. These variations hint at the profound influence of individual planting systems on the measured traits of chilli, with the Genotype (G), Environment (E), and G × E interactions showing high significance (P < 0.001) for all yield and yield-related attributes.

A key aspect of this genotype-environment interaction is highlighted by the differential positioning of the environmental markers across sectors on the biplot. This pattern sheds light on the unique performance of each genotype in their respective environments, which aligns with previous research underscoring the prevalence of gene-environment interactions across diverse traits. The differential positioning of the environmental markers across sectors further illuminates the unique performance of genotypes in respective environmental contexts, echoing previous research affirming the prevalence of gene-environment interactions across diverse traits^27,28^. Environmental factors such as nutrient availability, temperature, and humidity during the planting cycles also play a critical role in these interactions^29^. These observations of G × E interactions resonate with previous studies on the pronounced environmental impact on crop traits like those seen in chilli and wheat^15,30^. However, it’s important to note that variance analysis doesn’t entirely capture the G × E interactions’ complexity, with genotypic diversity inferred from a large sum of squares, suggesting significant differences among genotypic means and trait variability^5^.

Further, the study highlights the adaptability of specific genotypes across different planting systems. Genotypes G27 and G30, for instance, display superior yield across all planting systems, signifying their potential for widespread cultivation. These insights are crucial for enhancing agricultural practices, improving yields, and guiding effective selection. The GGE biplot’s utility becomes evident in this context, capturing a significant proportion of the genotype and genotype-by-environment variation for the studied traits, thereby effectively illustrating the genotype-environment interactions^10^. The AMMI2 analysis employed in our study proves instrumental in dissecting the complex genotype by environment interaction (GEI) in agronomy and plant breeding, consistent with prior research^30,31^. The significant variation explained by the first two principal components (PCs), amounting to 88.3%, 87.7%, 83.3%, and 83.2% for yield per plant, number of fruits, fruit’s length, and fruit’s width, respectively, highlights the importance of these components in capturing the GEI. This supports the argument that the performance of genotypes can vary significantly across different environments or planting systems^11,32^.

In the AMMI2 biplot model, positioning environmental markers and genotype vectors and identifying mega-environments partitioned into the first two PCs further demonstrated this GEI. This indicates that the environment, encompassing factors like nutrient availability and climatic conditions, is vital in influencing the genotypes’ performance across different planting systems^31^. Concurrently, our findings identify unique environmental sectors and genotypes showing high adaptability within these sectors, mirroring similar observations in previous research^28,33^. In chilli cultivation, various agronomic traits, such as the number of fruits, and fruit dimensions (length and width), significantly influence yield per plant. This yield is notably subject to variation with changes in planting environments, highlighting the relevance of genotype by environment (G × E) analysis in understanding environmental impacts on genotype expression^5^.

Genotype evaluation across diverse planting systems allows quantification and comparison of genotypic performance, aiding breeders in identifying superior performers for targeted selection and breeding efforts^11^. It also assists in discerning genotypes with broad or specific adaptability, as portrayed by the GGE biplot. The differential performance, as represented by genotypes G30, G27, and G7, which had the highest yield per plant, contrasts with genotypes G22 and G8, which yielded the least (Table 4). Likewise, genotypes G20 and G29 produced the most fruits, while G19 and G21 had the fewest. These variations underscore the significance of selecting appropriate genotypes to optimize yield across diverse environments, reaffirming the pivotal role of G × E interactions in shaping crop performance^15^. Therefore, a comprehensive understanding of G × E interactions is imperative for informed decisions on crop improvement strategies^30^.

The regression slope coefficient (b_i_) represents the change in the performance of a genotype in response to changes in environmental conditions to quantify the genotype’s responsiveness to different environments. A genotype with a ‘b_i_’ value less than 1.0 measures greater resistance to environmental change (above-average stability) and therefore increases the specificity of adaptability to low-yielding environments. In contrast, a cultivar with a ‘b_i_’ value of more than 1.0 is more prone to environmental changes and may not perform as well in challenging or unfavorable conditions. However, it may exhibit higher adaptability and productivity in environments that yield better results than those with a ‘bi’ value of exactly 1.0, present average stability, and be adaptable to all environments^34^. From the current finding for the yield per plant, the b_i_ value is ranged between − 1.15 and 0.04. The genotype is expressed as stable when it is shown the regression coefficient of unity (b_i_ = 1).

Although high mean yield is a desirable attribute but not an indicator of yield stability, thus the deviation from regression (S^2^_d_) value can help to quantify the portion of the variation that cannot be accounted for by the genotype–environment interaction^35^. The value explains the genotype stability across different planting systems or environmental conditions. A smaller value of S^2^_d_ suggests lower variability of observed genotype performance, indicating greater stability, and more consistency across diverse environments, suggesting that environmental fluctuations less influence their performance. On the other hand, larger values of S^2^_d_ indicate higher variability in genotype performance, indicating greater instability or sensitivity to environmental changes.

The univariate stability model is the commonly used method to measure and estimate stability^1,36^, including Shukla’s stability variance (σ^2^_i_), which is the method to measure the stability rather than the mean performance^37^, and Wricke’s ecovalence (W_i_^2^), which is the estimation on the contribution of each genotype to the G × E interaction sum of squares. The genotypes were considered stable if the genotype had low W_i_^2^ and σ^2^_i_ values.

The mean vs. stability diagram features an average-environment axis (AEA), depicted by a continuous single arrow line that represents the average performance of genotypes across the environment, which helps breeders to understand which genotypes perform better on average. On the other hand, the SVP line is typically drawn perpendicular to the AEA to assess genotype stability across different environments. Genotypes closer to the SVP line are more stable (their performance is more consistent across varying environments). In contrast, those further from the SVP line are less stable (their performance varies more across different environments)^38^.

Discriminative and representative: a discriminative analysis helps elucidate the performance variations among genotypes across various environmental conditions, their average performances, and stability.A discriminating ability of test location is measured by the length of location vectors, which is approximately the standard deviation within each location^38^. A representative location should effectively identify superior genotypes adapted to the whole environment or planting system. According to the result of this study, HydroStock and BioHydrogel were identified as superior planting systems due to their more discriminative and representativeness. Evaluating test locations based on their ability to discriminate and representativeness delivers important information about the efficiency of each location in recommending cultivars suited to specific or broad adaptation^39^.

## Conclusion

The study has effectively revealed the interactions between genotype and environment in chilli cultivation using various soilless planting systems. It demonstrated that these interactions significantly impact crop yield and other related traits, leading to the categorization of genotypes into three distinct groups: high-yielders, low-yielders, and stable performers. The study utilized advanced analytical tools like the GGE biplot and AMMI2 analysis to understand genotype performance and stability. Key findings include the identification of certain genotypes that excel in yield and fruit production, as well as those that show consistent performance across different environmental conditions. The research also revealed that among the planting systems evaluated, HydroStock and BioHydrogel were particularly effective in maximizing yield. These insights are crucial for guiding future crop breeding programs and optimizing agricultural practices. The study underscores the importance of selecting high-performing and stable genotypes and effective planting systems to enhance fruit productivity, contributing significantly to our understanding of genotype-environment interactions in fruit production. Further research is suggested to explore those high performing and stable genotypes under different environmental condition including the field trial to validate the findings and further expand our understanding og genotype–environment interactions.

## Data Availability

The datasets generated and analysed for the current study are available from the corresponding author on reasonable request.
